# A comparative study of ChIP-seq sequencing library preparation methods

**DOI:** 10.1186/s12864-016-3135-y

**Published:** 2016-10-21

**Authors:** Arvind Y. M. Sundaram, Timothy Hughes, Shea Biondi, Nathalie Bolduc, Sarah K. Bowman, Andrew Camilli, Yap C. Chew, Catherine Couture, Andrew Farmer, John P. Jerome, David W. Lazinski, Andrew McUsic, Xu Peng, Kamran Shazand, Feng Xu, Robert Lyle, Gregor D. Gilfillan

**Affiliations:** 1 Department of Medical Genetics, Oslo University Hospital and University of Oslo, Oslo, Norway; 2 Zymo Research Corp., 7062 Murphy Ave., Irvine, CA 92614 USA; 3 Takara Bio USA, Inc., 1290 Terra Bella Avenue, Mountain View, 94043 CA USA; 4 Mass. General Hospital, Mol. Biol., Harvard Medical School, 185 Cambridge St, CPZN 7250, Boston, 02114 MA USA; 5 Department Molecular Biology & Microbiology and Howard Hughes Medical Institute, Tufts University, 136 Harrison Avenue, Boston, 02111 MA USA; 6 Swift Biosciences, Inc., Suite 100, 58 Parkland Plaza, Ann Arbor, 48103 MI USA; 7 Rubicon Genomics, Inc., 4743 Venture Drive, Ann Arbor, 48108 MI USA; 8 Singapore Institute for Clinical Sciences, Agency for Science, Technology and Research (A*STAR), 117609 Singapore, Republic of Singapore; 9 Present address: Directed Genomics, 240 County Road, Ipswich, MA 01938 USA

**Keywords:** HTS, NGS, Low-input, Micro-ChIP, Chromatin immunoprecipitation

## Abstract

**Background:**

ChIP-seq is the primary technique used to investigate genome-wide protein-DNA interactions. As part of this procedure, immunoprecipitated DNA must undergo “library preparation” to enable subsequent high-throughput sequencing. To facilitate the analysis of biopsy samples and rare cell populations, there has been a recent proliferation of methods allowing sequencing library preparation from low-input DNA amounts. However, little information exists on the relative merits, performance, comparability and biases inherent to these procedures. Notably, recently developed single-cell ChIP procedures employing microfluidics must also employ library preparation reagents to allow downstream sequencing.

**Results:**

In this study, seven methods designed for low-input DNA/ChIP-seq sample preparation (Accel-NGS® 2S, Bowman-method, HTML-PCR, SeqPlex™, DNA SMART™, TELP and ThruPLEX®) were performed on five replicates of 1 ng and 0.1 ng input H3K4me3 ChIP material, and compared to a “gold standard” reference PCR-free dataset. The performance of each method was examined for the prevalence of unmappable reads, amplification-derived duplicate reads, reproducibility, and for the sensitivity and specificity of peak calling.

**Conclusions:**

We identified consistent high performance in a subset of the tested reagents, which should aid researchers in choosing the most appropriate reagents for their studies. Furthermore, we expect this work to drive future advances by identifying and encouraging use of the most promising methods and reagents. The results may also aid judgements on how comparable are existing datasets that have been prepared with different sample library preparation reagents.

**Electronic supplementary material:**

The online version of this article (doi:10.1186/s12864-016-3135-y) contains supplementary material, which is available to authorized users.

## Background

Immunoprecipitated DNA fragments enriched by chromatin immunoprecipitation can be analysed genome-wide by microarray hybridization (ChIP-chip), or by DNA sequencing (ChIP-seq). ChIP-seq confers a number of advantages [1], so is now the method of choice [2]. Although the use of sequencing as a readout for ChIP was first demonstrated using Sanger sequencing [3], the advent of high-throughput sequencing (HTS) has made widespread adoption of ChIP-seq possible [4–8].

In order to study the epigenome of specific cell types and small biopsy samples, epigenetic techniques are particularly driven to utilize low amounts of input DNA. Improvements to the ChIP immunoprecipitation itself allow locus-specific assays to be performed with as few as 100 cells [9–17], but genome-wide analysis requires more starting material. ChIP-chip protocols operating down to 1000-cell input amounts have been in use for the last decade [13, 15], employing amplification of the isolated ChIP DNA to microgram quantities to allow microarray hybridization. To use sequencing as a readout instead of arrays, less amplified material is required - typically tens of nanograms. Paradoxically, despite this lower final amount requirement, it has been more challenging to prepare small input amounts for sequencing. Due to multiple inefficient enzymatic steps and purifications required to ligate adapter sequences prior to sequencing, standard procedures for ChIP-seq sample preparation typically require 1–10 ng input DNA, limiting studies to the use of relatively large cell numbers (in the range of 100,000 or more – see for example references [17, 18]).

To meet the need for low-input library preparation for ChIP-seq, several techniques have been developed and refined (see Table 1 and references [19, 20]), allowing inputs down to 10 pg. The underlying principles vary, and include random-priming, adapter ligation, in vitro transcription and reverse transcription, extension of templates by terminal transferase and amplification from complementary homopolymer primers. However, PCR amplification is employed in all cases at some point during the procedure. It is not clear how comparable datasets generated by these different methods are, and to what extent they introduce bias in the results.

**Table 1:** Low-input library preparation methods tested in this study

Technique	Reference/Commercial supplier	Salient details	Reported DNA input range	Sequencing platform compatibility
Accel-NGS® 2S (Accel-2S)	Swift Biosciences, Inc.	5-step process of DNA repair, adapter ligation and PCR amplification. 5 purification steps.	0.01 – 1000 ng	Illumina
Modified Illumina method (Bowman)	Kingston Lab [36]	4 step procedure of end-repair, A-tailing, adapter ligation and PCR. 4 purification steps.	0.1 – 1000 ng	Illumina
HTML-PCR (HTML)	Camilli lab [37]	4-step procedure of end-repair, poly-C-tailing, poly-G-adapter oligo ligation and PCR. 4 purification steps.	0.01 – 100 ng	Illumina
SeqPlex™	Sigma Aldrich, Inc.	3-stage process of semi-random primed pre-amplification, PCR amplification, and primer removal. 2 purification steps.	0.1 – 1 ng	Agnostic (subsequent library prep required)
DNA SMART™ ChIP-Seq Kit (SMART)	Takara Bio USA, Inc.	5-step procedure of denaturation, dephosphorylation, T-tailing, DNA replication and template switching by reverse transcriptase and PCR. Compatible also with ssDNA. 1 purification step.	0.1 – 10 ng	Illumina
TELP	Xu lab [38]	5-step procedure of end-repair, poly-C-tailing, biotinylated primer extension, exonuclease digestion & streptavidin purification, adapter ligation and PCR. Compatible also with ssDNA. 3 purification steps.	0.025–25 ng	Illumina
ThruPLEX®	Rubicon Genomics	3 stage process of end repair, stem-loop adapter ligation and PCR amplification. 1 purification step.	0.05–50 ng	Illumina

In this study we compared the performance of seven diverse methods capable of handling DNA amounts down to ≤100 pg input (Table 1). Each procedure was performed on replicate DNA samples derived from a single large-scale H3K4me3 ChIP. To maximize the possibility that each technique was performed under optimum conditions by experienced laboratories, we distributed samples to the developers of the methods. Following preparation in the developer’s laboratories, sequencing libraries were returned to the Norwegian Sequencing Centre for sequencing and data analysis. In parallel, replicate datasets were generated by PCR-free sample preparation from the same ChIP sample, which created a reference with minimum possible bias. The methods were compared with respect to their generation of unmappable reads, duplicate reads, reproducibility, sensitivity (false negative rate) and specificity (false positive rate) relative to the reference dataset.

## Results

### ChIP, library preparation and sequencing

**Fig. 1:**
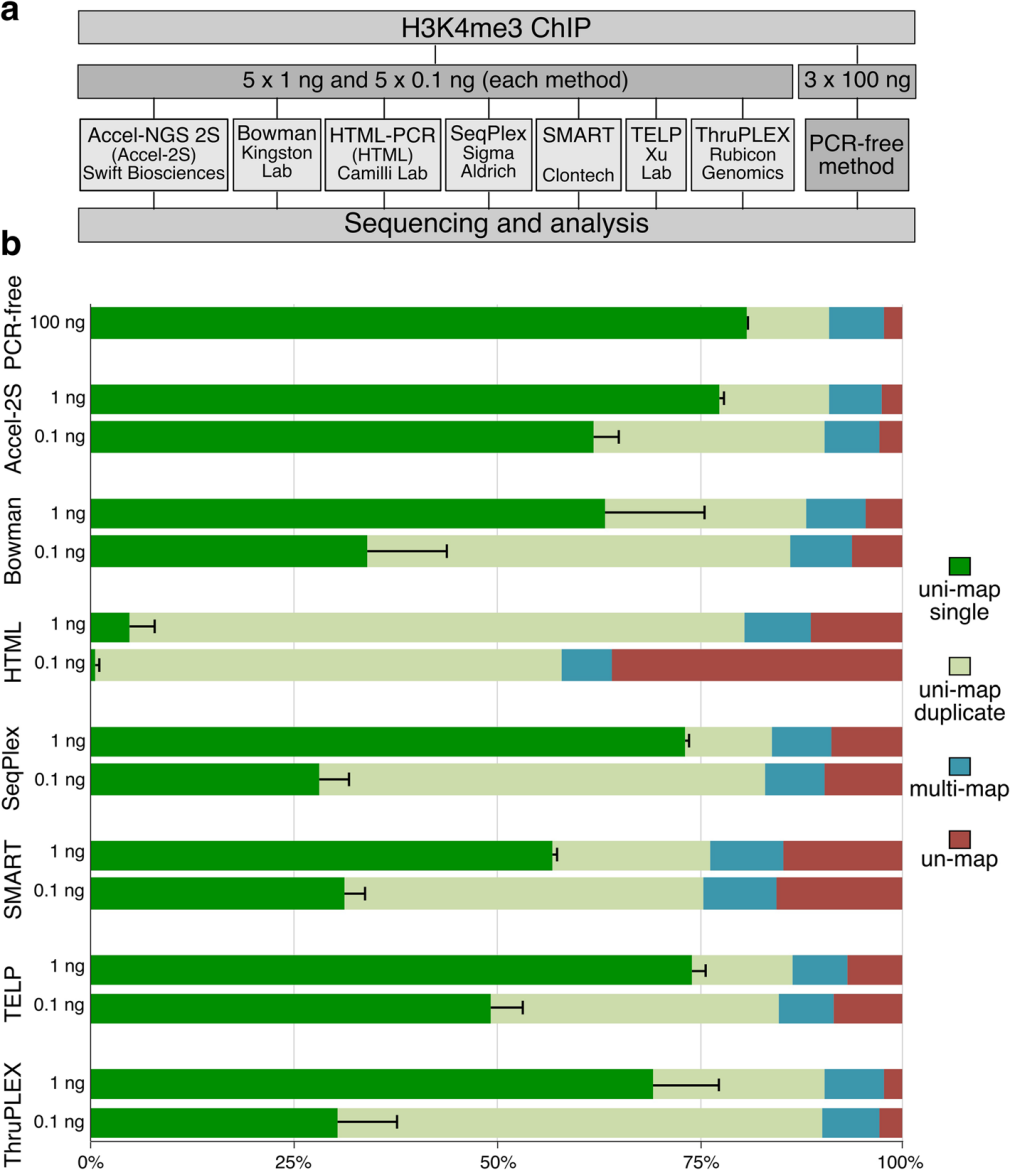
Experimental design and sequencing read mapping. **a** Experimental design overview. **b** Genomic mapping of sequence reads. The proportion of reads that were unmapped (red), those mapping to single genomic positions (*green*), and those mapping to multiple locations (repeats, in *blue*) are illustrated. Reads mapping to single genomic positions are broken down into reads present as a unique copy, and those present in two or more identical copies (duplicates). Results shown are the mean of 5 replicates for each method, using 25 million reads per replicate. Error bars show the standard deviation from the mean

Starting with 56 million HeLa cells, multiple ChIP reactions were performed using anti-H3K4me3 antibody. Material from all reactions was combined, yielding a single pool totalling 450 ng ChIP DNA on which all subsequent experiments were performed (Fig. 1a). To produce a reference dataset with the least possible technical bias, three replicate PCR-free libraries were prepared with 100 ng ChIP DNA apiece. The remaining ChIP DNA was divided into five lots, four of which were spiked with low amounts of DNA from other species (see methods) to control that replicate samples were processed separately through library preparation and not combined into a single pool to increase reproducibility. ChIP DNA (5 replicates each of 1 ng and 0.1 ng) was then shipped to participating laboratories for library preparation. Upon return of amplified libraries, the yield and size of the DNA was checked before samples were diluted and pooled for sequencing. Samples amplified by the SeqPlex method were at this point prepared for sequencing by performing PCR-free library prep, to avoid introducing additional amplification bias. The yield of each library produced, and the corresponding number of sequencing reads generated (range 28–74 million per sample), is detailed in Additional file 1: Table S1. Library sizes are documented in Additional file 1: Figure S1.

### Genomic read mapping

To compare the proportion of reads mapping uniquely, present in duplicate copies, and unmapped reads, all datasets were randomly down-sampled to 25 million reads (Fig. 1b). As expected, the highest proportion of uniquely mapping non-duplicate reads was seen in the PCR-free reference dataset. Amongst the amplified ChIP libraries, the Accel-2S samples had the highest proportion of unique reads at both 1 and 0.1 ng input levels. Broadly speaking, the different methods performed similarly on 1 ng input, but in several cases the number of unique reads dropped considerably with 0.1 ng input. The HTML samples had high levels of duplicate reads and unmappable reads. Attempts to improve mapping of HTML samples by trimming terminal bases that may have derived from the homopolymer tail added during preparation made little difference. HTML samples were therefore excluded from further analyses at this point.

### Analysis of library complexity and ChIP enrichment QC

**Fig. 2:**
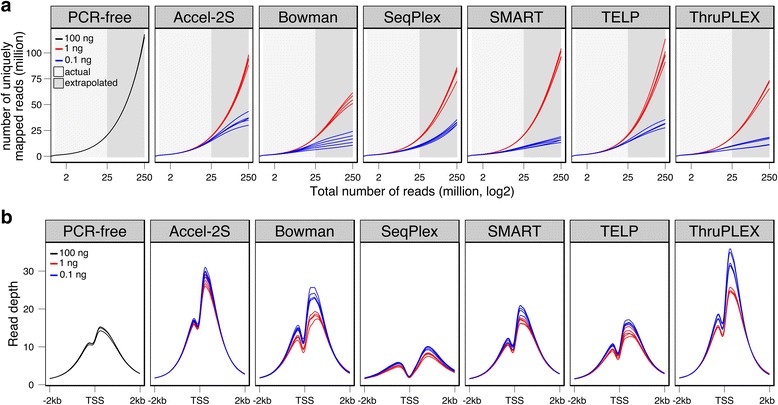
Library complexity and H3K4me3 ChIP signals intensity. **a** Library complexity curves generated using Preseq. Yield of uniquely mapping reads based on down-sampled data of 25 million reads. PCR-free (three replicates) method from 100 ng input is shown in black. Red and blue lines represent the five replicates used in 1 ng and 0.1 ng input across six low-input methods. Actual and extrapolated values are shaded in white and grey, respectively. X-axis is represented in log2 scale. **b** Read depth in a 4 kb window centered on known TSS. Colours as in (**a**)

To further examine library complexity, we employed the Preseq package [21]. Complexity curves for each library type are presented in Fig. 2a. The reference libraries showed the greatest complexity and least variation. At 1 ng input, all methods produced libraries of high complexity, with only minor differences in complexity and variation visible. All samples showed reduced complexity and greater variation at 0.1 ng input, with the greatest complexity retained by the Accel-2S, SeqPlex and TELP libraries.

In addition, we used NGS-QC [22] to assess the robustness of ChIP signal:noise to down-sampling of read depth. The QC-stamp and underlying QC-indicator scores for all samples are presented in Additional file 1: Table S2. In comparison to 1,612 H3K4me3 profiles held in the NGS-QC database, QC-stamp scores showed that all samples resembled closely existing H3K4me3 datasets, with Accel-2S showing the most consistent high scores at both 1 ng and 0.1 ng input levels.

To confirm the expected enrichment of H3K4me3 signals at promoters [4], we plotted read depth surrounding known transcription start sites (TSS). All methods showed the expected strong H3K4me3 enrichment surrounding TSS (Fig. 2b), with the characteristic drop in signal at the TSS itself caused by nucleosome eviction.

**Fig. 3:**
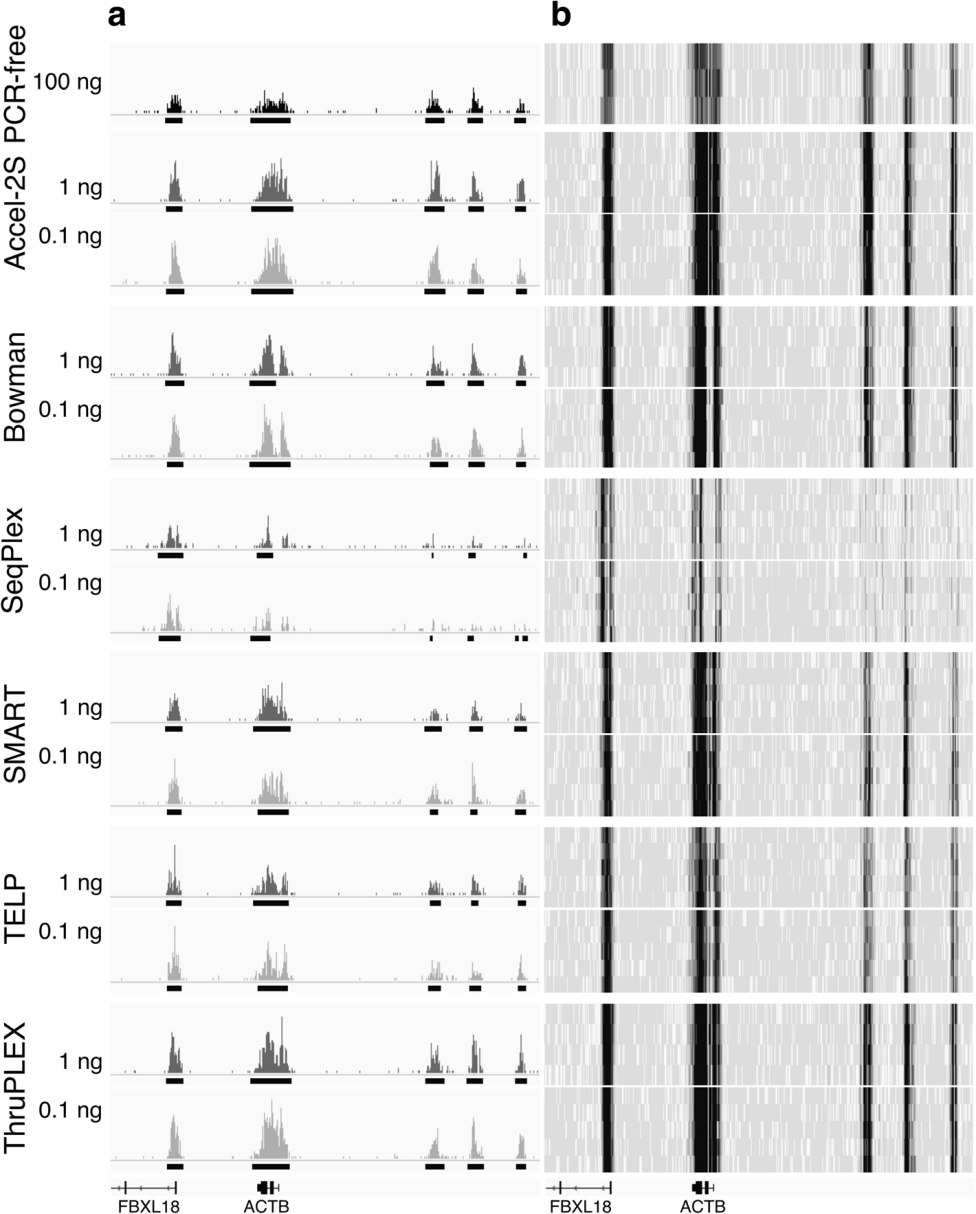
Data visualized in the Integrative Genomics Viewer [47]. **a** H3K4me3 distribution in an 80 kb genomic region. A single example PCR-free library is shown in black at the top, and libraries derived from 1 ng and 0.1 ng are shown in grey and light grey respectively.**b** Data for all libraries shown as heat maps of the same genomic region. Y-axis scale in all cases is read depth 0–28

Visual inspection (Fig. 3) confirmed that H3K4me3 binding can primarily be found at promoters, as expected from the literature [4] and Fig. 2b. In an ideal case, ChIP profiles amplified from low input amounts would closely resemble those produced from the PCR-free reference datasets. In all cases, the amplification of low input amounts can be seen to increase the signal at some genomic locations and lose signal at others, in relation to the PCR-free sample, with the effect most obvious on the samples amplified from 0.1 ng. These observations were confirmed in the full datasets (see Additional file 1: Figure S2). Nonetheless, the profiles generally matched the PCR-free dataset extremely well. The profiles most visibly distinct from the reference dataset were those generated by the SeqPlex method, which appear to have more background noise and less even coverage over H3K4me3 peaks, as supported by genome-wide sensitivity and specificity of peak calling (see below).

### Peak calling, sensitivity and specificity

To quantitatively assess the similarity of the various methods, peak calling was performed with MACS [23], using only uniquely-mapping, non-duplicate reads. To make the comparison between the methods as similar as possible, the same number of reads (5.5 million, a limit set by the sample with the lowest number) was used for all samples. Critically, despite using lower read numbers than available for most samples, peak calling approached saturation for all methods except SeqPlex (Additional file 1: Figure S3). Peak calling data is summarized in Table 2. Over 19,000 peaks were detected in each of the PCR-free datasets, and overlapping peaks (minimum overlap 1 bp) present in all three were chosen to define a set of 17,124 peaks as the reference to which all other methods were compared to measure sensitivity (false negatives) and specificity (false positives). Similar numbers of peaks (approx. 18–21,000) were called for all methods, with the exception of SeqPlex at 0.1 ng input, where > 35,000 peaks were called. The methods recorded sensitivity over 90 %, with the exception of SeqPlex, which had a lower sensitivity of 80 %. Specificity (off-target) rates showed a greater range of values. The highest sensitivity and specificity values were recorded for Accel-2S and ThruPLEX.

**Table 2:** Peak calling, sensitivity and specificity

Method	Input Amount (ng)	Mean number of peaks called	Mean number of reference peaks overlapped by sample peaks	Mean number of sample peaks not found in reference dataset	Mean sensitivity (% reference peaks detected)	Mean specificity (% method peaks found in reference dataset)
PCR-free	100	19 221 ± 157	17 124 ± 0	2 097 ± 157	100 %	89 %
Accel-2S	1	18 190 ± 123	16 574 ± 38	1 616 ± 115	97 %	91 %
	0.1	18 179 ± 124	16 505 ± 62	1 675 ± 103	96 %	91 %
Bowman	1	19 082 ± 334	16 096 ± 111	2 986 ± 228	94 %	84 %
	0.1	18 986 ± 365	16 155 ± 48	2 831 ± 384	94 %	85 %
SeqPlex	1	21 382 ± 265	13 612 ± 78	7 770 ± 255	79 %	64 %
	0.1	35 867 ± 3 861	13 905 ± 113	21 962 ± 3 902	81 %	39 %
SMART	1	17 906 ± 765	15 723 ± 99	2 182 ± 807	92 %	88 %
	0.1	19 893 ± 3 196	15 622 ± 166	4 271 ± 3 349	91 %	80 %
TELP	1	20 529 ± 1 592	15 528 ± 81	5 001 ± 1 641	91 %	76 %
	0.1	20 149 ± 1 619	15 370 ± 105	4 778 ± 1 683	90 %	77 %
ThruPLEX	1	18 377 ± 152	16 462 ± 36	1 916 ± 138	96 %	90 %
	0.1	18 015 ± 178	16 298 ± 44	1 717 ± 200	95 %	90 %

Peaks were called using MACS, using only 5.5 million uniquely mapping non-duplicate reads per sample. Peak regions present in all three PCR-free datasets (*n* = 17,124) were used as the reference dataset to which all other samples were compared to measure sensitivity and specificity. Data presented are mean +/− standard deviation

**Fig. 4:**
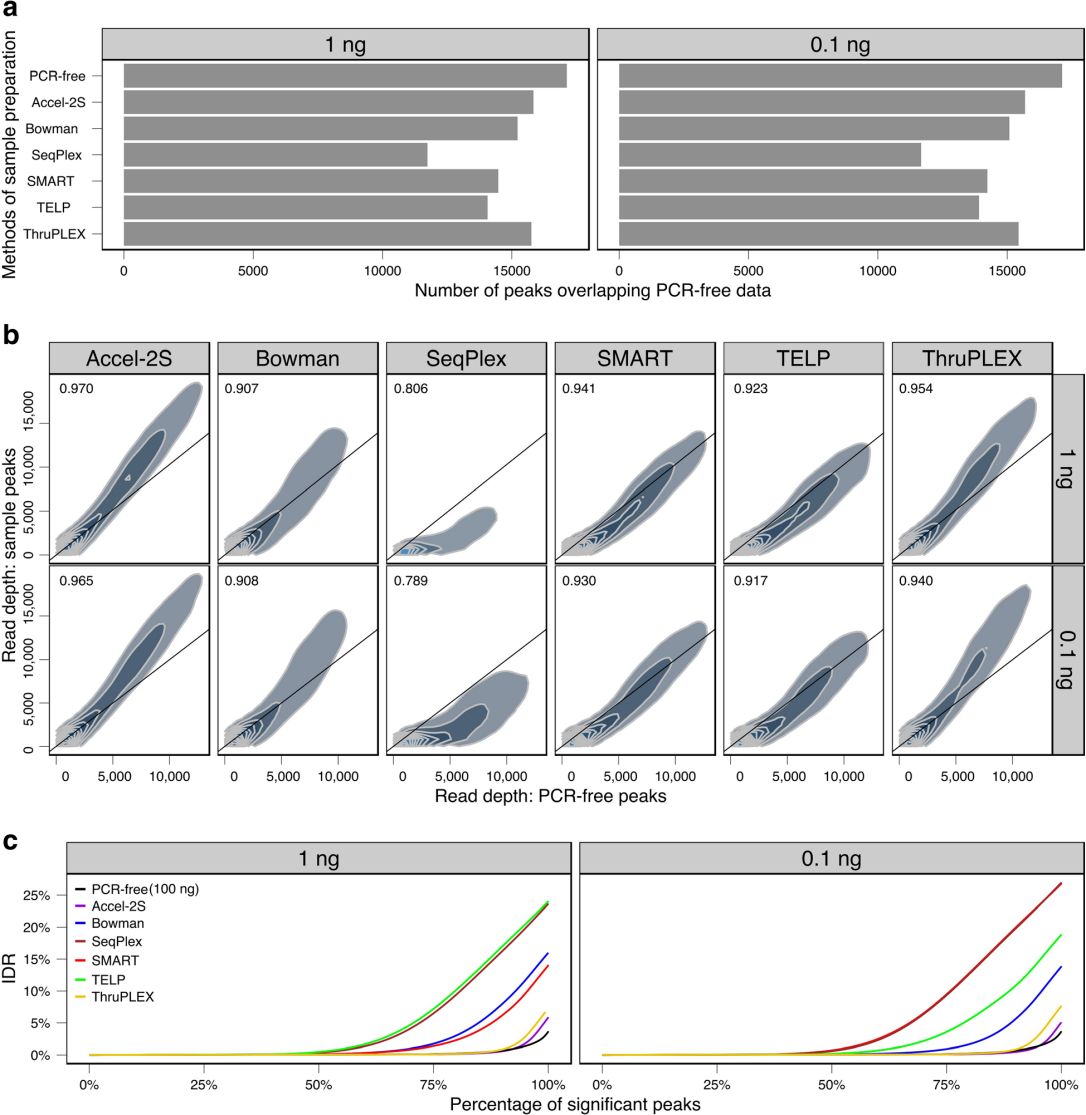
Correlation of peak calling and reproducibility of datasets. **a** Number of overlapping peaks found in all 5 replicates for the 1 ng and 0.1 ng input methods, and all three PCR-free replicates. **b** 2d density estimation of number of read bases within PCR-free peaks (present in all three replicates) against the number of reads bases within the same regions for all replicates of other methods. Data calculated using 5.5 million uniquely mapping non-duplicate reads for all methods, with 1 ng and 0.1 ng input level. Only reads mapping to peaks found in the PCR-free datasets were analyzed and included in correlation calculations. Spearman correlation coefficients for each method are given. Black line represents slope 1 and is provided for reference. **c** Irreproducible discovery rate (IDR) at different numbers of selected peaks, plotted at various IDR cutoffs for all methods. IDR for PCR-free (100 ng) method is shown in both 1 ng and 0.1 ng input panels for illustration

When examining peak overlaps under the most stringent conditions (peaks present in all five replicates for each method, and also in all three PCR-free reference replicates), it is evident that no two methods completely overlap (Additional file 1: Figure S4). The majority of peaks (11,020 at 1 ng input and 10,922 at 0.1 ng input) were detected by all methods. A number of peaks (3.1 % at 1 ng input, rising to 4.3 % at 0.1 ng input) were only found in the PCR-free datasets. The SeqPlex method stands out in failing to detect a significant number of peaks detected by all other methods (Fig. 4a).

To confirm that these observations held true with higher read numbers, we repeated peak calling on the 1 ng input datasets, which had a higher proportion of uniquely mapping reads, therefore allowing peak calling with 16 million uniquely mapping reads per sample (Additional file 1: Table S3). In this case, proportionally more peaks were called from the PCR-free dataset, resulting in a drop in sensitivity by a few percent for most techniques. Using this higher number of reads, the sensitivity of the Accel-2S, Bowman and ThruPLEX techniques was equal (94 %). The specificity of most methods was slightly increased relative to the peak calls made with 5.5 million reads, but their relative performance was unchanged.

To examine the consistency of results obtained by each method when performed with 1 or 0.1 ng input, we also examined the overlap between peaks called at both input amounts (Additional file 1: Table S4), a metric that reflects the scalability of each method, independent of the reference dataset. All methods except SeqPlex scored well, with Accel-2S showing the highest overlap of peak calls at the two input amounts.

Because the PCR-free reference dataset was prepared using Accel-2S reagents, it was of concern that this might bias peak calling on the low-input samples in favour of the Accel-2S method. We therefore compared peak calls to an independent, previously-published H3K4me3 dataset from HeLa cells, obtained using the same antibody used here, generated by the ENCODE consortium [24] (Additional file 1: Table S5). However, no significant changes in the relative performance of the low-input methods were seen, with Accel-2S and ThruPLEX retaining the highest sensitivity and specificity scores.

The above analyses of peak overlap do not take into account peak height. To assess the extent to which peak heights were correlated across the different methods, we counted the number of bases in mapped reads, and correlated these to the PCR-free reference dataset. The data (Fig. 4b) reveal, under both 1 and 0.1 ng input levels, strong correlations in all cases. Correlation coefficients rank (highest-lowest) in the following order: Accel-2S, ThruPLEX, SMART, TELP, Bowman and SeqPlex. It is worth noting the consistency of the correlations between the 1 ng and 0.1 ng datasets within each method, suggesting that input amount (within the ranges tested here) has less impact on results than the choice of library preparation method.

### Sources of variation

Of the peak calling metrics compared, the greatest differences were seen between methods when comparing specificity relative to the reference dataset. This suggested that the greatest source of variation between methods was in generation of noise/off-target amplification. To identify possible sources of this variation, we further examined the read depth, GC-content, width and MACS score of peaks called for each method (Additional file 1: Figure S5). These analyses suggested that SeqPlex in particular suffered from spurious amplification and preferential amplification of DNA with lower GC content. Unfortunately, none of the analyses illuminated a cut-off that could distinguish false-positive from genuine peaks to increase specificity, without drastically compromising sensitivity.

Three methods (SeqPlex, SMART and TELP) can also amplify single-stranded DNA, which may be generated by the high temperatures used during de-crosslinking of ChIP material [25], sonication, or in some cases by the protein of interest. It is possible that these methods can detect loci that would be missed by other techniques, including the PCR-free method. This would unfairly classify genuine H3K4me3 binding sites detected by these methods as false positive peaks. However, attempts to identify possible ssDNA peaks in our data did not yield convincing evidence of reproducibly detectable peaks (Additional file 1: Note S1). It should be noted that the H3K4me3 ChIP performed for this study was not expected to produce significant amounts of ssDNA to fully test this scenario, and we cannot exclude the possibility that ssDNA fragments amplified by SeqPlex, SMART and TELP methods contributed to peak calls in addition to dsDNA fragments from the same locations. Nonetheless, their failure to detect all PCR-free reference peaks suggests that overall they are not more sensitive, and may suffer from higher noise, which is also suggested by the fraction of reads found in peaks (FRiP) [26] (Additional file 1: Table S6).

### Reproducibility

Irreproducible discovery rate (IDR) [27] analysis was applied at the level of peak calling, to produce a curve that quantitatively evaluates consistency across replicates. High reproducibility produces a curve with a late transition to high IDR values. The number of significant peaks across the replicates for different IDR rates (0 to 30 % in 0.1 % increments) was calculated (Fig. 4c). All 17,124 peaks common to the three PCR-free reference replicates were identified as significant with only 4 % IDR. As can be expected, the IDR was higher for all other samples. At 1 ng input, Accel-2S and ThruPLEX are clearly superior to the other methods (all peaks significant at 6–7 % IDR). SMART and Bowman had intermediate performance (14–16 % IDR) and SeqPlex and TELP the poorest scores (24 %). A similar picture is seen at 0.1 ng input, but notably the SMART procedure performed poorly at the lower input amount (all peaks significant at 27 % IDR).

## Discussion

To the best of our knowledge this is the first thorough study of low-input HTS library construction techniques, comparing seven methods. A major strength of the current study was generation of sufficient ChIP material to allow the use of a PCR-free library preparation method, to which all other methods were compared. PCR has been identified as a major source of bias during sequencing library preparation [28], and can lead to the accumulation of duplicate and unmapped reads [17]. Generally, the lower the number cycles of amplification employed by the techniques studied here (see methods), the better their performance. This simple observation may both help direct future method development, and alert users of all methods to reduce amplification cycles to the minimum necessary to obtain sufficient DNA for sequencing.

The Accel-2S reagents from Swift Biosciences were chosen to construct the PCR-free libraries because they allow the lowest input amount (100 ng) currently possible without the use of PCR. The same Accel-2S reagents consistently produced the best results by all metrics in the current study. It must be borne in mind that the reference PCR-free dataset, to which all methods were compared throughout, was created with the same reagents, which may bias results in favour of Accel-2S. Nonetheless, when evaluating read mapping, library complexity and in comparison to previously published H3K4me3 datasets (which were not performed in relation to the PCR-free dataset), Accel-NGS 2S ranked highest, underscoring the quality of these reagents.

The ThruPLEX reagents from Rubicon Genomics scored a close second on the critical metrics of peak calling, peak strength correlation, and IDR. Notably, the efficient and single-tube protocol for these reagents also makes them an attractive choice.

The SMART reagents from Takara Bio USA, also an efficient single-tube protocol, do not appear to be as sensitive and specific as those discussed above, particularly at the lowest input amount used. However, they may offer additional sensitivity if single-stranded DNA molecules are also present in the ChIP material. The TELP protocol may offer similar benefits of sensitivity regarding ssDNA, although the current study was not designed to thoroughly test this possibility. It is worth noting that modifications to the relatively new TELP and SMART procedures, currently undergoing additional development, may further improve performance.

The Bowman method, representing a highly optimized version of the standard Illumina library preparation method, also performed extremely well. When considering that many labs using the standard reagents struggle to obtain good quality libraries with 1 ng input DNA, the recommended minimum, labs that wish to continue using standard reagents and protocols may consider implementing the modifications contained within the Bowman method.

The SeqPlex method from Sigma Aldrich did not perform as well as the other methods, demonstrating a relatively low sensitivity and high false positive rate with 0.1 ng input. Nonetheless, it is the only low-input method tested that is sequencing platform agnostic.

With the exception of the HTML protocol, all the methods studied herein achieved good or extremely good metrics on the parameters examined given the challenging input amounts. The HTML method was not developed for ChIP samples but has been applied to sequence microbial genomes from low input amounts. Further development of the method may improve its performance with ChIP samples.

Importantly, it should be noted that, by this study´s design, library preparations were performed by different researchers, which may have influenced the results obtained. Effects of reagent age may also affect the results reported. Further optimization of each method for the particular DNA sample (ChIP protein, modification, FAIRE etc.), or customization of data analysis, may potentially narrow performance differences observed here. Furthermore, input control DNA (non-immunoprecipitated ChIP DNA) was not employed in this study, to emphasise differences between the methods. However, such input controls may compensate for amplification artefacts that result in false positive peaks, and could potentially make the results of the different techniques more comparable.

It should also be noted that this study was not exhaustive, as the originators of two methods (LinDA and nano-ChIP-seq [19, 20]) that met inclusion criteria did not participate. The recent adaptation of transposase-based tagmentation for use in ChIP may provide an additional alternative method, although library preparation in this case is performed prior to cross-link reversal [29]. There have been a further slew of methodological improvements demonstrating ChIP-seq even down to single cells. These advances entail library construction on chromatin before immunoprecipitation [30, 31], the use of carrier proteins or RNA [32], optimized lysis and fragmentation conditions [33], and microfluidics [34, 35]. However, these studies all employed reagents and methods equivalent to those tested here for sequencing library amplification. The choice of library preparation reagents therefore remains of paramount importance for data consistency and quality.

## Conclusions

We compared the performance of seven low-input library preparation methods on 1–0.1 ng ChIP material with regard to amplification fidelity, reproducibility, sensitivity and specificity relative to an unamplified “gold standard”. The Accel-NGS 2S reagents consistently achieved top ranking, but several other reagents also performed well. That several reagents achieved similar results is reassuring, as it suggests that many existing datasets (prepared with a wide variety of reagents) may be largely comparable. We nonetheless observed stronger differences in results between reagent types, than was seen when comparing data derived from the same reagents prepared with 1 or 0.1 ng input. Whilst we consider using equal input amounts of samples an important criterion to obtain optimum results, we urge researchers to choose their library preparation reagents carefully, optimise amplification conditions, and as a minimum use the same reagents within a study to maximise consistency.

## Methods

### Cell culture and chromatin immunoprecipitation

HeLa cells were purchased from ATCC and monitored during growth to check cell morphology by microscopy and ensure absence of Mycoplasma contamination by PCR assay. Cells were grown in Advanced DMEM (Dulbecco's Modified Eagle Medium) media at 37 °C. Cells were cross-linked with formaldehyde at 1 % final concentration for 7 min at room temperature, and chromatin prepared using the Zymo-Spin ChIP kit (Zymo Research Corp., Irvine, CA) and anti-H3K4me3 antibody (Millipore 07–473; lot #2430389), following manufacturer’s instructions. Sonication was performed at high power setting for 40 cycles (30 s on, 30 s off) using a Bioruptor Plus (Diagenode Inc., Denville, NJ), yielding a modal fragment size of 180 bp (Additional file 1: Figure S6). A total of 56 million cells were processed using 56 Zymo-Spin ChIP reactions and the resulting ChIP DNA concentrated and combined into a single pool using ChIP DNA Clean & Concentrator (Zymo Research Corp., Irvine, CA).

### Spike DNA preparation

DNA spiked into distributed ChIP samples was isolated from *Campylobacter jejuni*, *Escherichia coli*, *Saccharomyces cerevisiae* and *Staphylococcus haemolyticus* and were gifts from users of the Norwegian Sequencing Centre. One microgram genomic DNA from these organisms was sonicated to modal size 200 bp using a Covaris E220 instrument (Covaris Inc., Woburn, MA), and diluted for blending with ChIP DNA to approximately 1 %.

### Distribution of DNA samples to participants

Each participant received five replicates containing 1 ng ChIP DNA (0.2 ng/μl in 10 mM Tris pH 8) and five replicates containing 0.1 ng ChIP DNA (0.02 ng/μl in 10 mM Tris pH 8). Participants also received a further 2 ng ChIP DNA and 10 ng sonicated input DNA for the purposes of optimizing library preparation prior to handling the replicate samples destined for sequencing. To minimize any possible effects of adapter sequences on ligation and/or amplification efficiency, it was required that the different methods use the same five indexed adapter sequences during library preparation where possible.

### Illumina sequencing library preparation

#### PCR-free libraries

100 ng ChIP DNA was used as input to the Accel-NGS® 2S DNA Library Kit for Illumina (Swift Biosciences, Ann Arbor, MI). Manufacturer’s instructions were followed (protocol version 04291444), with the exception that SPRIselect (Beckman Coulter, Brea, CA) bead cleanup steps 1 and 2 used 1.2 volumes of beads, in order to maximize recovery of the 175 bp ChIP DNA.

#### Accel-NGS® 2S (Accel-2S)

Accel-NGS 2S was performed according to manufacturer’s instructions (version 04291444), with 1.4 volumes of SPRIselect following steps 1, 2, and 3. Following step 4, a double-sided SPRIselect bead clean-up was performed: 0.64 volumes beads (32 μl beads added to 50 μl reaction volume, 5 min incubation, supernatant transferred to new tube) followed by a 1.0 volume second addition (18 μl beads added to transferred supernatant). For the post-PCR SPRI step, 1 volume of beads was used. For library amplification, 10 cycles of PCR were used for 1 ng samples and 14 cycles for 100 pg samples.

#### Bowman method

The Bowman method was performed according to reference [36]. For 1 ng input samples, 1 μL of 0.125 μM adapters were used in the ligation reaction and 14 PCR cycles were used to amplify the library. For 0.1 ng input samples, 1 μL of 0.1 μM adapters were used in the ligation reaction and 15 PCR cycles were used to amplify the libraries.

#### HTML-PCR (HTML)

HTML-PCR was performed according to reference [37], employing 30 cycles of PCR for all samples.

#### SeqPlex™

SeqPlex Enhanced DNA Amplification Kit (SEQXE) reagents were used following manufacturer´s instructions. Amplification was performed for 19 and 23 cycles for 1 ng and 0.1 ng samples respectively. Following return of SeqPlex-amplified material to the Norwegian Sequencing Centre, 250 ng each sample was used as input for PCR-free library preparation using Accel-NGS 2S reagents, as detailed above.

#### DNA SMART™ ChIP-Seq Kit (SMART)

SMART was performed according to manufacturer´s instructions, using 15 PCR cycles for 1 ng input and 18 PCR cycles for 0.1 ng input samples. Final library purification was performed using Option 4 (0.9 volumes SPRI beads).

#### TELP

TELP was performed as described [38], with the exceptions that all samples were subjected to end repair before entering the TELP procedure, and 15 μl magnetic beads were used instead of 8 μl. Furthermore, only a single round of PCR was performed in 30 μl volume for 15 cycles (1 ng input) and 18 cycles (0.1 ng input samples).

#### ThruPLEX® DNA-seq (ThruPLEX)

The manufacturer´s protocol for the ThruPLEX DNA-seq kit was followed, employing a total of 10 and 15 cycles PCR for the 1 ng and 0.1 ng input samples respectively.

### DNA concentration and size measurements

The concentration and size of ChIP and amplified library DNA used in the study was controlled using fluorescence (Qubit: Thermo Fisher Scientific, Waltham, MA) and electrophoresis (Bioanalyzer 2100: Agilent Technologies, Santa Clara, CA). High Sensitivity reagents were used in both cases according to manufacturer’s instructions.

### High-throughput sequencing

Sequencing (50 bp single reads) was performed on an Illumina HiSeq 2500 using v4 cluster generation and sequencing reagents (Illumina, San Diego, CA). Five indexed libraries were sequenced per lane so that each library could expect to obtain in the region of 40 million total reads. To avoid any possible lane bias during sequencing, samples were pooled such that no two libraries from the same method were run together in the same lane, and no two methods consistently run together in the same lane. An exception was made for the HTML libraries, which require a custom sequencing primer, thus all 10 HTML libraries were run together on two lanes. For the two lanes that contained HTML-PCR libraries, the custom sequencing primer olj719 (ACACTCTTTCCCTACAGCTGCGAGGGGGGG) was added to the HP10 reagent well at 0.5 μmol. The three PCR-free reference libraries were assigned two lanes, thus the entire experiment occupied two flow cells (16 lanes) of a single sequencing run. Two replicates were excluded (a single 1 ng input sample each from Bowman and ThruPLEX methods) due to concerns regarding sample integrity following shipment. Sequencing quality evaluation was performed using FastQC v0.11.3 [39] to calculate initial performance metrics.

### Data analysis

#### Base calling and QC

Initial image analysis and base calling were performed using RTA v1.18.61 (HCS v2.2.58; Illumina, San Diego, CA) on an Illumina HiSeq 2500. Bcl2fastq v1.8.4 (Illumina, San Diego, CA) was used to demultiplex the data into individual samples based on the indexes used during the library preparation. Since over 90 % of the reads had sequence quality scores over Q33 and the lowest mean quality score per sample (per base) was 27, reads were not trimmed based on quality and the raw sequence data was used for further analysis. As per manufacturer’s recommendations, the first three bases were trimmed from reads derived from the SMART method.

#### Read mapping

Raw sequence data were mapped to *C. jejuni*, *E. coli*, *S. cerevisiae* and *S. haemolyticus* genome sequences using BBMap v34.56 [40] to confirm that the replicates were processed separately during library preparation. Raw reads were mapped to the human reference genome (release hg19/GRCh37) using BWA v0.7.12-r1039 with default settings. Prior to mapping, the first three bases were trimmed from reads derived from the SMART method, as recommended by the manufacturer. Further analyses were carried out using BEDtools v2.20.1 [41], deepTools [42], Picard tools v1.112 [43] and SAMtools v1.2 [44] wherever applicable. Mapped data were down-sampled to 25 million reads and metrics such as unmapped, single and multi-mapped (both unique and duplicates) were collected using Picard. Reads mapping to a single location without duplicates were extracted and further down-sampled to 5.5 million and 16 million reads.

#### Preseq

Down-sampled 25 million read datasets were subjected to lc-curve using Preseq v1.0.2 [21] with –quick parameter and without bootstrapping for confidence intervals. Data was extrapolated up to 250 million, which is the current limit of data that can be sequenced using one full lane in Illumina HiSeq using lc-extrap.

#### NGS-QC

NGS-QC v150310.1.21526 [22] was run using the Galaxy online tool [45] with 25 million reads per sample. Human genome hg19, target molecule HeK4me3, background subtraction and clonal reads removal options were used.

#### Peak calling

Peak calling was performed with MACS v1.4.2 [23] using bandwidth equal to the modal size of sheared chromatin (−−bw = 180). Peak calling was only based on reads mapping to a single location, excluding duplicates. Published H3K4me3 ChIP-seq datasets from the ENCODE consortium (Bernstein lab) used for comparison were obtained from the Gene Expression Omnibus [46], accession number GSM733682.

#### IDR

IDR was calculated for MACS scores across five replicates in each method using R (R v3.2.1) package idr v1.2 [27] using default parameters.

## Additional file

Additional file 1: Supplementary Figures, Note and Tables. (PDF 1325 kb)

## Abbreviations

ChIP: Chromatin immunoprecipitation
H3K4me3: Histone H3 Lysine 4 trimethylation
HTS: High throughput sequencing
NGS: Next generation sequencing
