# Supplementary material for: A comparative study of ChIP-seq sequencing library preparation methods
Source: BMC Genomics. 2016 Oct 21;17:816. doi: 10.1186/s12864-016-3135-y (PMC5073829; doi:10.1186/s12864-016-3135-y)
Supplement: Additional file 1: — Supplementary Figures, Note and Tables. (PDF 1325 kb) [file 12864_2016_3135_MOESM1_ESM.pdf]

Additional File 1. Supplementary Figures 1-6, Tables 1-6 and Note 1.

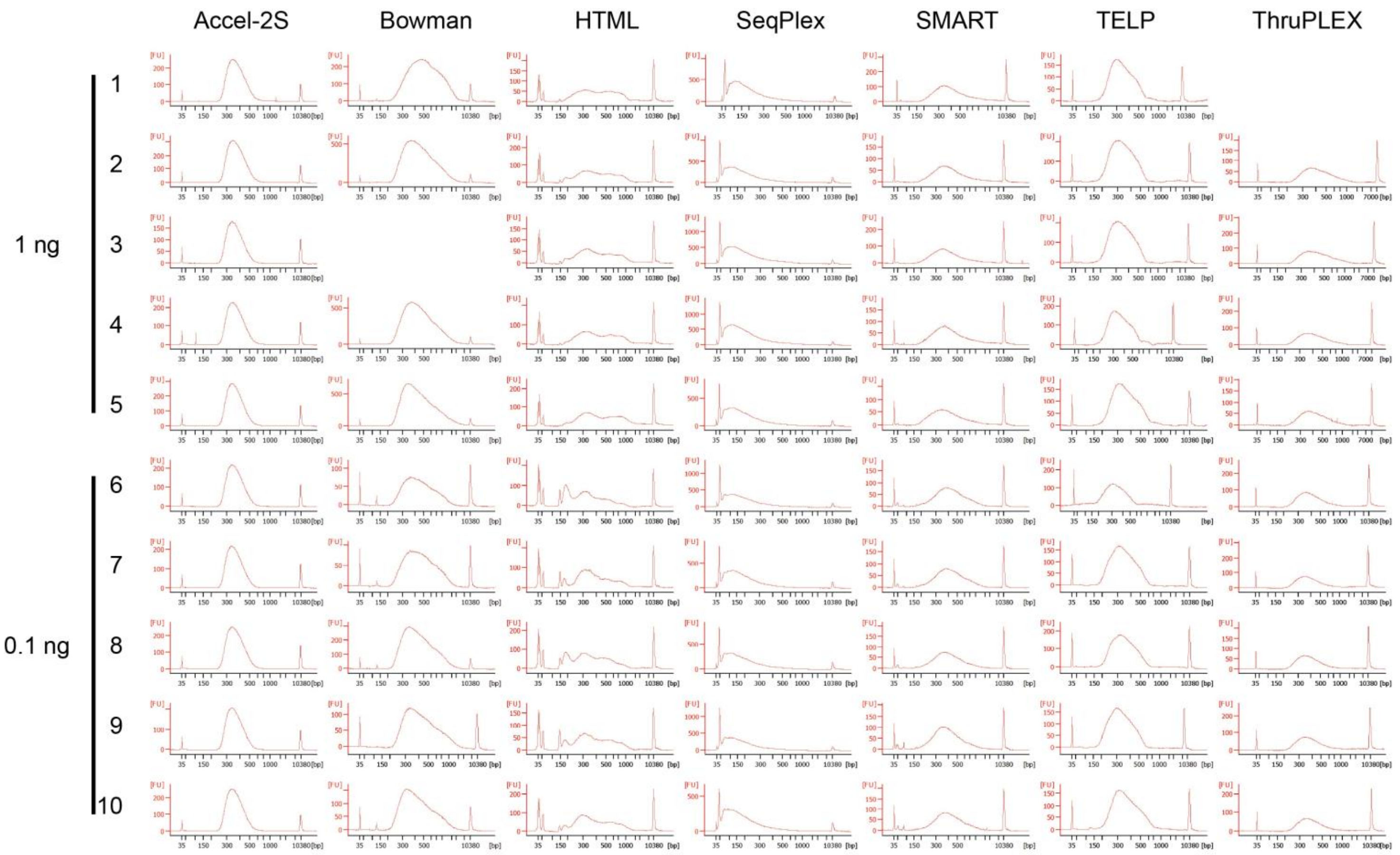

Figure S1. Library sizes determined by Agilent 2100 Bioanalyzer. Note that SeqPlex samples are shown following SeqPlex amplification, but prior to Illumina library adapter ligation, which was performed by a PCR-free method that attaches partially single-stranded adapters, causing aberrant migration in electrophoresis. SeqPlex amplified products shown above are therefore approximately 120 bp shorter than the final library products that were sequenced. All other libraries are shown at their full length that was sequenced.

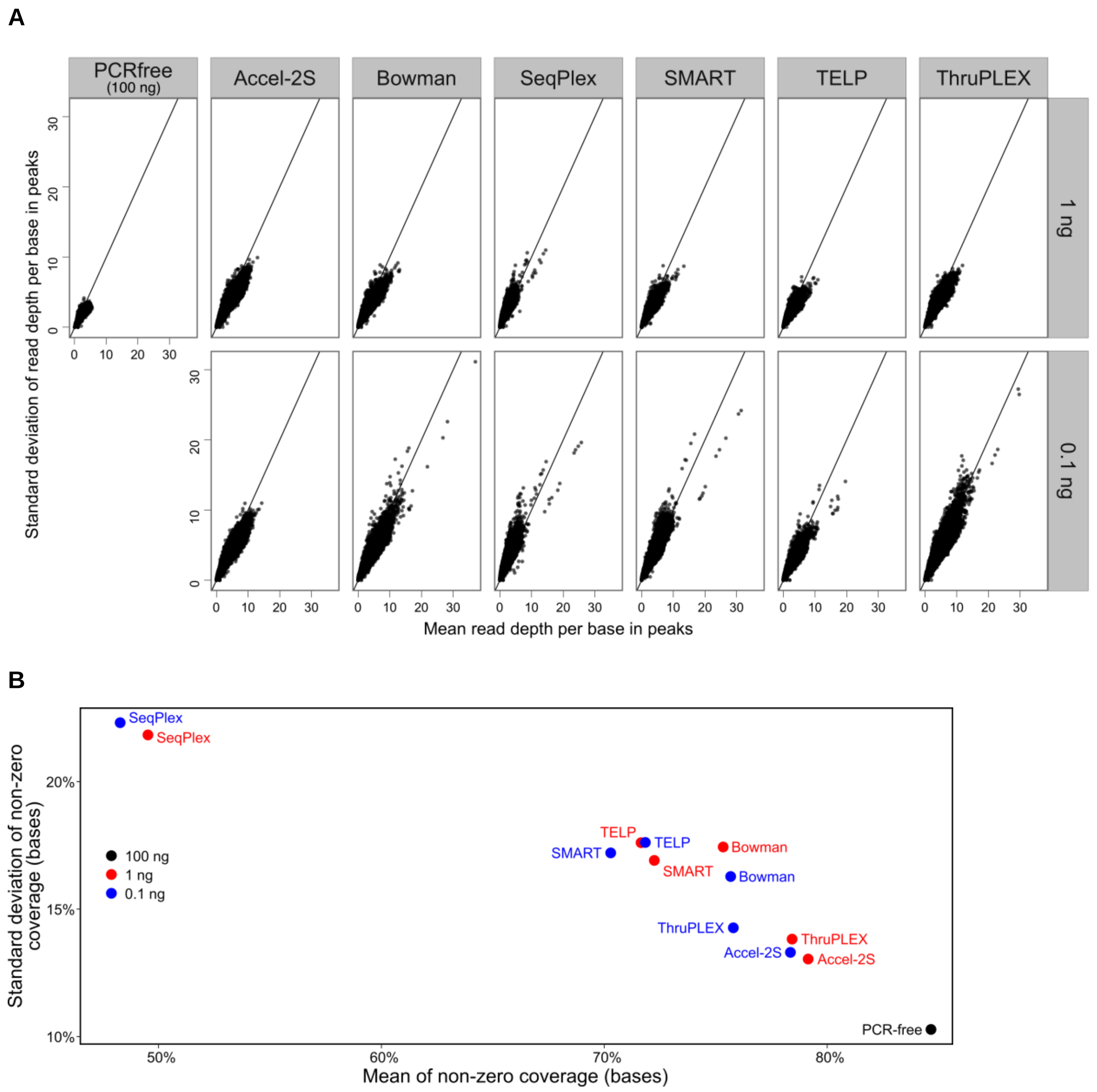

Figure S2. Low-input library preparation results in amplification of some sequences and loss of others. (**A**) Mean read depth is increased in amplified samples relative to the PCR-free dataset in H3K4me3 peaks, suggesting selective amplification of some sequences. Peak regions present in all three PCR-free datasets (n=17 124 peaks called by MACS 1.4.2, see Table 2 in manuscript) were used as the reference dataset to which all other samples were compared. Within reference peak regions, mean read depth per base, and standard deviation between replicates, was calculated. Figure shows mean and SD. Data source was 5.5 million uniquely mapping non-duplicate reads per sample. (**B**) Gaps (regions of zero read coverage) in peaks. Within reference peak regions, mean coverage (bases to which one or more reads were mapped) and standard deviation from all 5 replicates within each group were calculated. Figure shows mean and SD. Highest coverage and lowest SD were seen with the PCR-free samples, and more gaps and greater variation in the amplified samples, suggesting the failure to efficiently amplify some sequences.

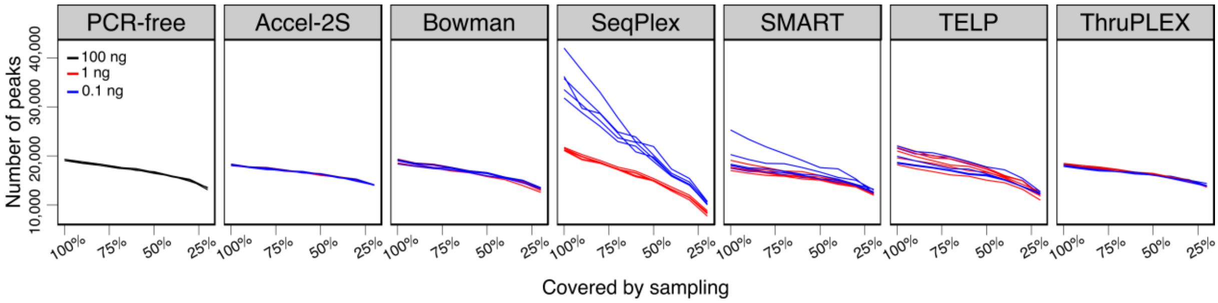

Figure S3. Saturation of peak calling as reads are randomly discarded. Data from MACS 1.4.2 (--diag option), starting with 5.5 million uniquely mapping reads per sample (100%).

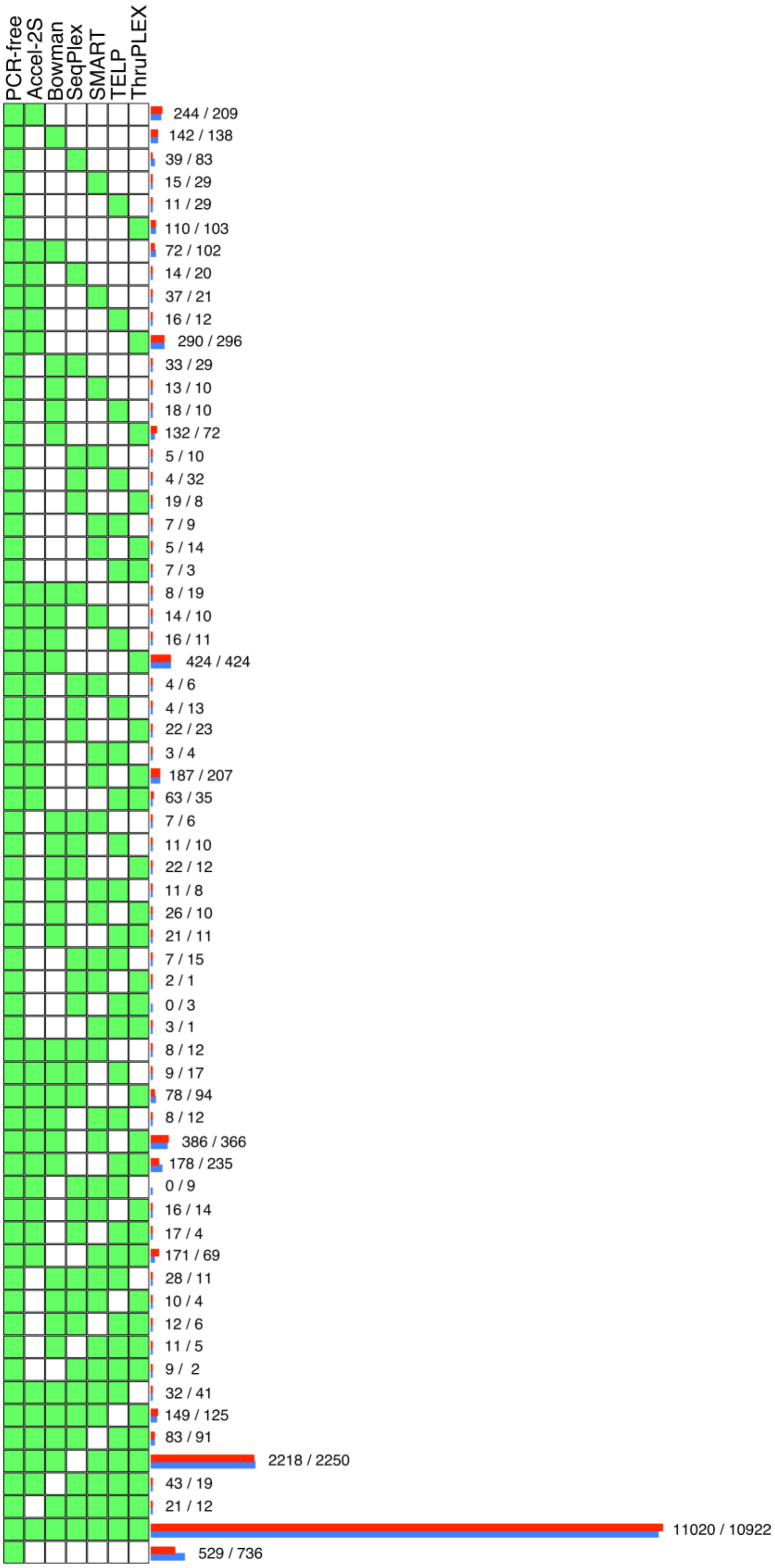

Figure S4. Summary of peak overlaps in all possible combinations of library types (green = present, white = absent). Peak numbers common to all 5 replicates for the 1 ng (red bars) and 0.1 ng (blue bars) low input methods, with peaks common to all three PCR-free replicates, are detailed.

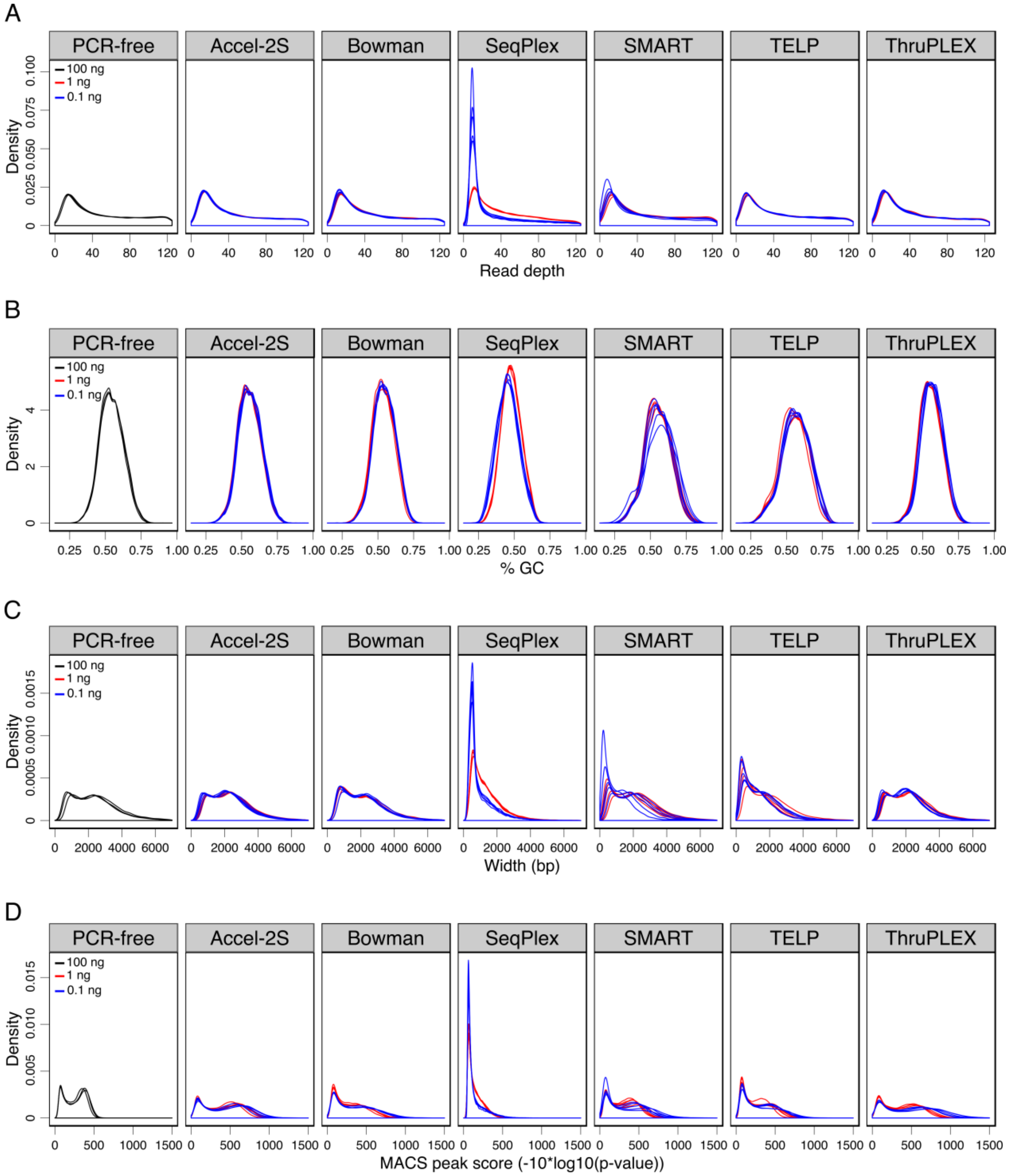

Figure S5. Density plots of read depth, GC content, width and MACS score within peaks. PCR-free (three replicates) method from 100 ng input is shown in black. Red and blue lines represent the five replicates used in 1 ng and 0.1 ng input across six low-input methods. (**A**) Number of reads mapping to peaks identified by MACS within individual samples using 5.5M reads. The high number of peaks with low read depth generated by SeqPlex at 0.1 ng input suggests that spurious amplification / noise was responsible for the low specificity seen in this case. (**B**) GC-content within peaks. GC-content of peak regions was similar for all methods, with two exceptions: SMART and TELP methods showed a broader range of GC content (particularly at low GC-content) than other methods, which may be related to the amplification of ssDNA not detected by other methods, although this remains to be

tested. SeqPlex showed a general shift to lower GC-content relative to other methods, more pronounced in the 0.1 ng input samples, which may indicate preferential amplification (amplification bias). (**C**) Peak width. (**D**) Peak MACS score. In both the case of peak width and MACS score, a bimodal distribution was visible, suggesting a dichotomy of broad, deep-coverage, high-confidence versus narrow, low-coverage and low-confidence peak calls. This was confirmed by further analysis (i.e. low-confidence peak calls are low-coverage narrow peaks, data not shown). Particularly regarding peak width, Accel-2S, Bowman and ThruPLEX closely match the results seen with the PCR-free reference, whilst SMART and TELP showed slightly increased numbers of low-coverage and narrow peaks and greater sample-sample variability. With respect to peak MACS scores, low-input amplification by all methods resulted in a proportion of peaks obtaining higher scores than those produced by the PCR-free method, reflecting increased read depth and a corresponding increase in peak score at sites favourably amplified by PCR. This conclusion is supported by the fraction of reads found in peaks (FRiP – see Supplementary Table 6): Accel-2S, ThruPLEX and Bowman methods present a higher percentage of mapped reads within regions called as peaks (averages 56-65%) than the PCR-free reference (average 47%), and all 0.1 ng input samples have higher FRiP values than 1 ng samples.

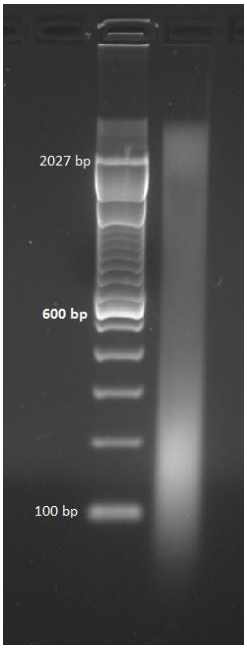

Figure S6. Chromatin shearing. 500 ng chromatin (pre-immunoprecipitation), following cross-link reversal, run on a 1.5 % TBE agarose gel, is shown beside 100 bp ladder.

Note 1

Attempt to identify peaks potentially derived from ssDNA/ unique to the SeqPlex, SMART and TELP methods. When calling peaks with 5.5 M reads per sample, a number of peaks were detected in all ten replicates of the SeqPlex (n = 490), SMART (n = 283) and TELP (n = 300) methods, that were absent in any of the PCR-free replicates. Such peaks could conceivably be derived from ssDNA, which these methods can amplify, that other methods (including the PCR-free reference) would fail to detect. Alternatively, they could simply represent peaks that were detected more efficiently following amplification by the above methods relative to the PCR-free standard. In an attempt to distinguish these possibilities, we repeated peak calling on the PCR-free datasets using the maximum number of available uniquely mapping reads (88-97 million reads per sample), to maximize detection of otherwise weak H3K4me3 signals in the PCR-free dataset. Using this higher number of reads to call peaks in the PCR-free datasets: In the case of SMART and TELP, no candidate ssDNA peaks (present in all 10 replicates yet absent in the PCR-free dataset) were retained. In the case of SeqPlex, approximately 100 peaks common to all ten replicates remained, but were all found to map to genomic regions of low complexity. These observations suggested that neither SeqPlex, SMART nor TELP reproducibly detected peaks exclusively derived from ssDNA in the H3K4me3 ChIP material tested here.

Group Name	Sample	Library conc. (ng/µl)	% of lane	Mean % of lane yield	Total reads passing filter	Mean # reads	SD reads	Mapped reads	Single map unique reads	Single map duplicate reads	Multiple map unique reads	Multiple map duplicate reads
Accel 1ng	Accel-2S-1	5.5	20.5	17.8	45111678	37804853	6235516	43742904	31623991	9150464	2588891	379558
	Accel-2S-2	5.4	21.4		42355272			41143315	30047992	8316459	2438758	340106
	Accel-2S-3	3.4	13.9		31472814			30542444	23121528	5391821	1816916	212179
	Accel-2S-4	4.1	14.7		31431712			30572400	23349795	5165548	1846340	210717
	Accel-2S-5	4.7	18.6		38652791			38090593	28084549	7434184	2269257	302603
Accel 100pg	Accel-2S-6	4.4	17.7	18.3	35868769	39556218	3686968	34666891	19367044	12955023	1924126	420698
	Accel-2S-7	3.5	19.7		40039103			38730813	18737073	17405045	2033058	555637
	Accel-2S-8	4.2	17.2		39866020			38412787	20308825	15488914	2105820	509228
	Accel-2S-9	4.7	17.5		36745744			35663670	19918921	13343081	1968504	433164
	Accel-2S-10	5.5	19.4		45261455			44473948	24396462	17060531	2442177	574778
Bowman 1ng	Bowman-1	8.6	20.5	19.2	44344327	42110111	7285990	41845936	24494745	13979892	2736070	635229
	Bowman-2	17.9	23.2		50994774			49020804	28936954	16250541	3103541	729768
	Bowman-4	16.0	17.3		39150019			37548188	23537699	11045798	2449329	515362
	Bowman-5	19.0	15.9		33951322			33286531	22454579	8244467	2207660	379825
Bowman 100pg	Bowman-6	2.6	17.0	18.8	37899374	40363595	7086131	34017694	5606939	25732675	1461211	1216869
	Bowman-7	7.1	17.3		35060492			33461180	11383054	19448168	1838256	791702
	Bowman-8	4.2	21.2		43264701			39952137	7883368	28895263	1838449	1335057
	Bowman-9	4.6	22.1		51352441			49107583	15092684	30169948	2570350	1274601
	Bowman-10	4.9	16.3		34240968			32491391	9341947	20529147	1731513	888784
HTML 1ng	HTML-1	26.6	22.0	19.6	53754701	47813612	3703738	48709558	1738574	42461944	1200245	3308795
	HTML-2	29.4	19.6		47820017			37145584	870379	32886836	725578	2662791
	HTML-3	26.2	18.2		44433155			41722931	643371	37194541	670234	3214785
	HTML-4	30.8	19.7		48087623			40708874	1009294	36043625	824545	2831410
	HTML-5	31.4	18.4		44972564			43625138	3040970	36632847	1457359	2493962

HTML 100pg	HTML-6	19.8	17.9	19.6	40493475	44206645	3983365	22276757	178265	19697338	293367	2107787
	HTML-7	23.0	19.6		44283415			32433677	105644	29278918	200930	2848185
	HTML-8	21.4	18.0		40697218			17448619	370421	15381847	351317	1345034
	HTML-9	25.6	20.1		45347711			32022362	200290	28843794	296876	2681402
	HTML-10	24.4	22.2		50211406			39669348	215302	35632034	422688	3399324
PCRfree (100 ng)	PCRfree-1	3.2	61.4	64.7	156079842	164602649	11749925	152712933	88285431	53747628	8266129	2413745
	PCRfree-2	3.3	62.8		159721730			155987107	89960341	55109819	8427709	2489238
	PCRfree-3	3.3	70.0		178006374			174074785	97424849	64459208	9272790	2917938
SeqPlex 1ng	SeqPlex-1	5.2	14.0	17.1	29898156	36327433	9312398	25440306	16472976	6236729	2265587	465014
	SeqPlex-2	4.5	16.9		35067334			29881377	18806508	7941784	2565248	567837
	SeqPlex-3	5.2	15.8		34192852			28992281	18523433	7398913	2516482	553453
	SeqPlex-4	4.3	15.1		30033429			25605633	16539377	6354194	2244002	468060
	SeqPlex-5	4.5	23.9		52445394			45302788	24200592	16330413	3671977	1099806
SeqPlex 100pg	SeqPlex-6	4.3	14.9	16.4	31464022	35407596	8634940	26479293	8428767	15260772	1915280	874474
	SeqPlex-7	4.7	21.5		50161573			42373751	11451957	26471076	2834326	1616392
	SeqPlex-8	4.7	14.6		32525616			27415028	9126164	15344903	2046368	897593
	SeqPlex-9	4.4	13.7		27888008			23627833	9027194	12119527	1805529	675583
	SeqPlex-10	4.8	17.2		34998759			29596458	8080975	18357591	2052514	1105378
SMART 1ng	SMART-1	10.1	19.5	17.7	40436769	37903325	3582936	36932174	27375176	6605304	2555839	395855
	SMART-2	11.9	18.5		39929618			36311646	27406607	5987587	2535927	381525
	SMART-3	9.7	15.9		31631176			28761244	22209384	4247757	2034762	269341
	SMART-4	11.7	17.6		38660816			35185582	26922330	5431581	2466604	365067
	SMART-5	7.3	17.2		38858248			35790094	26696460	6219610	2494141	379883
SMART 100pg	SMART-6	3.6	18.3	20.7	42610592	45305107	5040671	38121955	7198763	27857661	1681245	1384286
	SMART-7	5.1	22.9		50884498			45697949	9591067	32462620	2083120	1561142
	SMART-8	4.5	18.9		38446433			34756616	8351106	23677087	1656958	1071465
	SMART-9	5.6	22.2		45254053			40977840	9966952	27793735	1955196	1261957

	SMART-10	4.3	21.3		49329959			44783622	7265798	34002203	1814361	1701260
TELP 1ng	TELP-1	2.6	27.6	26.8	62410689	56910318	11149304	58299183	41739486	12177939	3636511	745247
	TELP-2	3.3	34.5		73691822			68732635	43948088	19678791	4119351	986405
	TELP-3	2.7	22.6		46793490			43572392	30296064	10083377	2687509	505442
	TELP-4	2.6	22.6		48705230			44787749	31672060	9731966	2860374	523349
	TELP-5	2.7	26.7		52950359			49980187	34709768	11481063	3180370	608986
TELP 100pg	TELP-6	2.6	19.8	23.8	45876853	51995442	5509638	41528979	15855585	22536818	2234838	901738
	TELP-7	2.8	24.2		50875952			46656752	20126066	23034444	2571377	924865
	TELP-8	2.4	22.4		52305453			47727427	14889853	29287368	2355777	1194429
	TELP-9	2.3	22.5		50044732			45716023	17186109	25072157	2453500	1004257
	TELP-10	3.1	30.0		60874218			56253345	18779487	33198583	2888992	1386283
ThruPLEX 1ng	ThruPLEX-2	14.1	19.6	18.7	44220953	40280965	3309543	42818851	28425846	11249638	2700897	442470
	ThruPLEX-3	12.1	17.0		36268935			35226411	24398740	8213153	2288050	326468
	ThruPLEX-4	10.2	19.1		39530622			38526658	25375302	10317384	2438547	395425
	ThruPLEX-5	12.4	19.0		41103350			40517279	27310398	10212864	2590173	403844
ThruPLEX 100pg	ThruPLEX-6	4.5	15.7	18.4	36286893	40215283	6614980	35191492	6006229	26627366	1396369	1161528
	ThruPLEX-7	10.5	23.9		36489833			35093845	10341919	22142379	1757414	852133
	ThruPLEX-8	10.7	14.8		50278253			48777537	10423081	34746970	2146818	1460668
	ThruPLEX-9	9.7	19.6		34452468			33444970	10172001	20785390	1684773	802806
	ThruPLEX-10	5.1	17.8		43568966			42790079	6247989	33412904	1589222	1539964

Table S1. Individual library and sequencing yields.

**Sample**	**QC Stamp**	**denQC (2.5%) s50**	**simQC (2.5%) s90/s50**	**denQC (5%) s50**	**simQC (5%) s90/s50**	**denQC (10%) s50**	**simQC (10%) s90/s50**
Accel2S-1	AAB	0.405366	4.260956	1.227305	2.03606	2.125244	1.378158
Accel2S-2	AAB	0.409938	4.219871	1.219755	2.046993	2.126106	1.377169
Accel2S-3	AAB	0.429274	4.311664	1.260052	2.080289	2.187176	1.396675
Accel2S-4	AAB	0.42284	4.141007	1.24667	2.017802	2.142873	1.374477
Accel2S-5	AAB	0.417131	4.153028	1.235474	2.019877	2.120703	1.37989
Accel2S-6	AAB	0.395051	4.811642	1.278468	2.154829	2.310676	1.391279
Accel2S-7	AAB	0.40638	4.8385	1.317869	2.256349	2.42347	1.428433
Accel2S-8	AAB	0.385775	4.864932	1.252879	2.279556	2.303504	1.441956
Accel2S-9	AAB	0.397739	4.750966	1.267804	2.15291	2.296203	1.39092
Accel2S-10	AAB	0.394923	4.745306	1.252061	2.151448	2.248587	1.39885
Bowman-1	ABB	0.246412	6.539536	0.907523	2.670973	1.898535	1.533538
Bowman-2	AAB	0.26763	6.148778	0.960884	2.534696	1.931091	1.499517
Bowman-4	AAB	0.265841	6.138008	0.952801	2.544163	1.922344	1.506257
Bowman-5	AAB	0.281105	5.92351	0.98368	2.498188	1.960539	1.494372
Bowman-6	BBB	0.171698	10.416718	0.855802	3.750126	2.39845	1.757626
Bowman-7	ABB	0.224356	7.573543	0.95021	3.117364	2.208037	1.600598
Bowman-8	BBB	0.189049	9.077225	0.886979	3.421181	2.306735	1.7191
Bowman-9	ABB	0.248233	6.816912	0.980974	2.986804	2.176437	1.592384
Bowman-10	BBB	0.199061	8.087209	0.871369	3.267864	2.151118	1.73208
HTML-1	CCC	0.054983	26.041754	0.345278	8.390292	1.532629	2.671959
HTML-2	CCC	0.037265	36.69231	0.245804	11.563412	1.212538	3.304374
HTML-3	DDC	0.030508	39.680553	0.188347	13.194601	0.983259	3.559793

HTML-4	CCC	0.041189	33.232559	0.258147	10.891157	1.271578	3.135844
HTML-5	BBC	0.084645	18.711485	0.508186	6.034525	1.922177	2.198224
HTML-6	DDD	0.016293	27.85	0.078615	13.336788	0.397558	4
HTML-7	CDD	0.025018	18.333332	0.104242	9.160001	0.423522	3.35865
HTML-8	DDD	0.018702	41.533333	0.092885	18.416107	0.561672	4.35849
HTML-9	DDD	0.019079	29.31111	0.093273	13.645455	0.456191	4.060409
HTML-10	DDD	0.023331	28.796875	0.115195	12.848101	0.532231	4.019863
PCRfree-1	AAB	0.379023	4.513313	1.180856	2.113914	2.089914	1.408961
PCRfree-2	AAB	0.384898	4.457674	1.177873	2.133056	2.096019	1.410582
PCRfree-3	AAB	0.378288	4.525338	1.180815	2.111303	2.093151	1.405868
SMRT-1	ABB	0.233723	6.335361	0.85076	2.561771	1.712783	1.508027
SMRT-2	ABB	0.226987	6.570771	0.840193	2.613346	1.717932	1.519981
SMRT-3	ABB	0.232949	6.32417	0.844182	2.558635	1.70665	1.502211
SMRT-4	ABB	0.226007	6.613716	0.834391	2.631577	1.711541	1.525393
SMRT-5	ABB	0.225203	6.45785	0.8318	2.573584	1.68282	1.506028
SMRT-6	BBB	0.106341	11.758329	0.565312	4.08439	1.692988	1.824869
SMRT-7	BBB	0.135433	9.669978	0.657021	3.593417	1.773075	1.751294
SMRT-8	BBB	0.134374	9.998158	0.675724	3.559475	1.820379	1.727811
SMRT-9	BBB	0.147161	9.111913	0.701044	3.409955	1.824947	1.716249
SMRT-10	BBB	0.119916	11.106431	0.617195	3.953251	1.799987	1.792505
SeqPlex-1	BBC	0.107335	16.572895	0.518396	6.132714	1.646627	2.333242
SeqPlex-2	BBC	0.109376	15.924675	0.523603	5.911224	1.645902	2.271954
SeqPlex-3	BBC	0.107461	16.247557	0.519643	5.977759	1.637573	2.292029

SeqPlex-4	BBC	0.111763	15.824541	0.532633	5.907787	1.665495	2.285106
SeqPlex-5	BBC	0.106635	16.888292	0.517948	6.189856	1.660914	2.330759
SeqPlex-6	BCC	0.093109	19.039728	0.489588	8.054779	1.943962	3.02234
SeqPlex-7	BCC	0.095427	18.66375	0.493959	8.09508	1.952787	3.046642
SeqPlex-8	BCC	0.084043	21.059965	0.453615	8.839476	1.894575	3.171917
SeqPlex-9	BCC	0.096959	18.022091	0.499018	7.832595	1.81233	2.549455
SeqPlex-10	BCC	0.088354	20.280235	0.470398	8.570266	1.927523	3.133729
TELP-1	ABB	0.211235	6.472151	0.802559	2.599313	1.656032	1.524841
TELP-2	ABB	0.21915	6.800483	0.819653	2.664391	1.694813	1.535172
TELP-3	ABB	0.227604	6.579879	0.847087	2.615872	1.742807	1.515468
TELP-4	ABB	0.209242	6.918703	0.7935	2.69359	1.655803	1.53655
TELP-5	ABB	0.212497	7.090452	0.807732	2.750311	1.703918	1.557263
TELP-6	BBB	0.152396	8.12993	0.667222	3.321194	1.604055	1.641565
TELP-7	BBB	0.16892	7.59806	0.70876	2.963268	1.60524	1.560746
TELP-8	BBB	0.151395	8.269901	0.675984	3.280001	1.63157	1.6206
TELP-9	BBB	0.159422	7.805429	0.679768	2.998426	1.572633	1.552943
TELP-10	BBB	0.166329	7.66254	0.702129	2.975948	1.618162	1.550296
ThruPlex-2	AAB	0.370049	4.929999	1.167474	2.246477	2.129825	1.442457
ThruPlex-3	AAB	0.36013	4.991673	1.149517	2.255111	2.115672	1.440855
ThruPlex-4	AAB	0.373001	4.867506	1.183341	2.208975	2.146692	1.42917
ThruPlex-5	AAB	0.375977	4.843253	1.165341	2.243934	2.136523	1.438264
ThruPlex-6	ABB	0.24285	8.22016	1.096535	3.145392	2.724134	1.597851
ThruPlex-7	AAB	0.27261	6.487333	1.091099	2.693894	2.316952	1.500779
ThruPlex-8	AAB	0.27136	6.62124	1.098657	2.725343	2.359952	1.502552

ThruPlex-9	AAB	0.273332	6.500999	1.081632	2.723792	2.323443	1.499245
ThruPlex-10	BBB	0.233383	8.324311	1.068787	3.135932	2.645676	1.606392

Table S2. NGS-QC indicators.

Method	Input Amount (ng)	Mean number of peaks called	Mean number of reference peaks overlapped by sample peaks	Mean number of sample peaks not found in reference dataset	Mean sensitivity (% reference peaks detected)	Mean specificity (% method peaks found in reference dataset)
PCR-free	100	23,434 ± 274	20,933 ± 0	2,501 ± 274	100 %	89 %
Accel-2S	1	21,196 ± 340	19,636 ± 162	1,561 ± 184	94 %	93 %
Bowman	1	22,421 ± 375	19,595 ± 101	2,826 ± 299	94 %	87 %
SeqPlex	1	36,338 ± 1,457	17,345 ± 57	18,994 ± 1,482	83 %	48 %
SMART	1	21,014 ± 572	18,638 ± 103	2,376 ± 628	89 %	89 %
TELP	1	24,338 ± 688	18,666 ± 144	5,672 ± 760	89 %	77 %
ThruPLEX	1	21,708 ± 99	19,677 ± 51	2,032 ± 72	94 %	91 %

Table S3. Peak calling with 16 M uniquely mapping reads in 1 ng input samples

Method	Combined total number of peaks (1 ng input)	Combined total number of peaks (0.1 ng input)	Number of 1 ng peaks found in 0.1 ng input	Percent 1 ng peaks detected in 0.1 ng input
Accel-2S	20843	20962	19563	93,9
Bowman	21821	22705	20287	93,0
SeqPlex	33119	97655	26968	81,4
SMART	20750	23605	19199	92,5
TELP	22799	21907	20502	89,9
ThruPLEX	20777	20473	19273	92,8

Table S4. Overlap of peaks called for each method at 1 ng and 0.1 ng input.

Method	Input Amount (ng)	Mean number of peaks called	Mean number of ENCODE peaks overlapped by sample peaks	Mean number of sample peaks not found in ENCODE dataset	Mean sensitivity (% ENCODE peaks detected)	Mean specificity (% method peaks found in ENCODE dataset)
ENCODE	unknown	24680 ± 1848	21291 ± 0	3389 ± 1848	100 %	87 %
Accel 2S	1	18190 ± 123	14432 ± 32	3758 ± 102	68 %	79 %
	0.1	18179 ± 124	14387 ± 68	3793 ± 78	68 %	79 %
Bowman	1	19082± 334	14509 ± 47	4574 ± 294	68 %	76 %
	0.1	18986 ± 365	14471 ± 65	4515 ± 350	68 %	76 %
SeqPlex	1	21382 ± 265	12700 ± 75	8682 ± 254	60 %	59 %
	0.1	35867 ± 3861	13213 ± 75	22654 ± 3866	62 %	37 %
SMART	1	17906 ± 765	14000 ± 80	3906 ± 781	66 %	78 %
	0.1	19893 ± 3196	13926 ± 77	5967 ± 3237	65 %	71 %
TELP	1	20529 ± 1592	13926 ± 84	6603 ± 1656	65 %	68 %
	0.1	20149 ± 1619	13725 ± 93	6424 ± 1659	64 %	68 %
ThruPLEX	1	18377± 152	14521 ± 43	3857± 142	68 %	79 %
	0.1	18015 ± 178	14224 ± 56	3791 ± 209	67 %	79 %

Table S5. Peak calling with 5.5 M uniquely mapping reads, compared to an ENCODE H3K4me3 dataset. ENCODE dataset was compiled from two replicate samples.

Group Name	Sample	Reads	Reads within peaks	FRiP	Mean FRiP	SD FRiP
Accel 1ng	Accel-2S-1	5500001	3474212	0.63	0.65	0.01
	Accel-2S-2	5499796	3507174	0.64		
	Accel-2S-3	5499538	3658319	0.67		
	Accel-2S-4	5499584	3615720	0.66		
	Accel-2S-5	5499875	3543626	0.64		
Accel 100pg	Accel-2S-6	5498777	3670663	0.67	0.66	0.01
	Accel-2S-7	5498221	3720480	0.68		
	Accel-2S-8	5499678	3624255	0.66		
	Accel-2S-9	5499463	3642985	0.66		
	Accel-2S-10	5499941	3590177	0.65		
Bowman 1ng	Bowman-1	5500101	2779907	0.51	0.52	0.01
	Bowman-2	5499945	2854996	0.52		
	Bowman-4	5499609	2886667	0.52		
	Bowman-5	5499697	2947393	0.54		
Bowman 100pg	Bowman-6	5499698	3395187	0.62	0.59	0.02
	Bowman-7	5501557	3211747	0.58		
	Bowman-8	5500826	3287364	0.60		
	Bowman-9	5499726	3264258	0.59		
	Bowman-10	5502090	3146658	0.57		
PCRfree (100 ng)	PCRfree-1	5499785	2645771	0.48	0.47	0.01
	PCRfree-2	5499066	2642428	0.48		
	PCRfree-3	5499117	2538591	0.46		
SeqPlex 1ng	SeqPlex-1	5499807	1370803	0.25	0.24	0.01
	SeqPlex-2	5498025	1366011	0.25		
	SeqPlex-3	5498106	1327347	0.24		

	SeqPlex-4	5499763	1395833	0.25		
	SeqPlex-5	5500109	1259644	0.23		
SeqPlex 100pg	SeqPlex-6	5501256	1760888	0.32	0.31	0.01
	SeqPlex-7	5501161	1664943	0.30		
	SeqPlex-8	5502198	1637192	0.30		
	SeqPlex-9	5501616	1710224	0.31		
	SeqPlex-10	5501064	1797914	0.33		
SMART 1ng	SMART-1	5499821	2554346	0.46	0.46	0.01
	SMART-2	5499788	2443787	0.44		
	SMART-3	5500184	2525034	0.46		
	SMART-4	5499862	2480233	0.45		
	SMART-5	5499917	2549131	0.46		
SMART 100pg	SMART-6	5500701	2433387	0.44	0.49	0.03
	SMART-7	5501756	2737172	0.50		
	SMART-8	5501890	2788555	0.51		
	SMART-9	5501744	2701959	0.49		
	SMART-10	5500317	2818748	0.51		
TELP 1ng	TELP-1	5497335	2146561	0.39	0.40	0.02
	TELP-2	5497312	2073288	0.38		
	TELP-3	5500008	2388140	0.43		
	TELP-4	5499853	2138841	0.39		
	TELP-5	5499061	2142693	0.39		
TELP 100pg	TELP-6	5500054	2419524	0.44	0.43	0.01
	TELP-7	5499688	2355129	0.43		
	TELP-8	5500083	2391592	0.43		
	TELP-9	5498872	2387873	0.43		
	TELP-10	5497986	2344310	0.43		

ThruPLEX 1ng	ThruPLEX-2	5500170	3357889	0.61	0.63	0.01
	ThruPLEX-3	5499984	3358186	0.61		
	ThruPLEX-4	5501002	3409441	0.62		
	ThruPLEX-5	5499905	3346614	0.61		
ThruPLEX 100pg	ThruPLEX-6	5500296	3933336	0.72	0.68	0.02
	ThruPLEX-7	5502315	3702538	0.67		
	ThruPLEX-8	5502098	3728176	0.68		
	ThruPLEX-9	5501948	3683665	0.67		
	ThruPLEX-10	5500581	3866571	0.70		

Table S6. Fraction of reads in peaks (FRiP).
